# Recommendations for exercise adherence measures in musculoskeletal settings: a systematic review and consensus meeting (protocol)

**DOI:** 10.1186/2046-4053-3-10

**Published:** 2014-02-10

**Authors:** Melanie A Holden, Kirstie L Haywood, Tanzila A Potia, Melanie Gee, Sionnadh McLean

**Affiliations:** 1 Arthritis Research UK Primary Care Centre, Keele University, Keele ST5 5BG, UK; 2 Royal College of Nursing Research Institute, Warwick Medical School, Warwick University, Coventry CV4 7AL, UK; 3 Faculty of Health and Wellbeing, Collegiate Campus, Sheffield Hallam University, Sheffield S10 2BP, UK; 4 Centre for Health and Social Care Research, Collegiate Campus, Sheffield Hallam University, Sheffield S10 2BP, UK

**Keywords:** Acceptability, Adherence, Consensus, Exercise, Measurement, Musculoskeletal disorders, Physical activity, Quality, Systematic review

## Abstract

**Background:**

Exercise programmes are frequently advocated for the management of musculoskeletal disorders; however, adherence is an important pre-requisite for their success. The assessment of exercise adherence requires the use of relevant and appropriate measures, but guidance for appropriate assessment does not exist. This research will identify and evaluate the quality and acceptability of all measures used to assess exercise adherence within a musculoskeletal setting, seeking to reach consensus for the most relevant and appropriate measures for application in research and/or clinical practice settings.

**Methods/design:**

There are two key stages to the proposed research. First, a systematic review of the quality and acceptability of measures used to assess exercise adherence in musculoskeletal disorders; second, a consensus meeting. The systematic review will be conducted in two phases and reported in accordance with the Preferred Reporting Items for Systematic Reviews and Meta-Analyses (PRISMA) guidelines to ensure a robust methodology. Phase one will identify all measures that have been used to assess exercise adherence in a musculoskeletal setting. Phase two will seek to identify published and unpublished evidence of the measurement and practical properties of identified measures. Study quality will be assessed against the COnsensus-based Standards for the selection of health Measurement Instruments (COSMIN) guidelines. A shortlist of best quality measures will be produced for consideration during stage two: a meeting of relevant stakeholders in the United Kingdom during which consensus on the most relevant and appropriate measures of exercise adherence for application in research and/or clinical practice settings will be sought.

**Discussion:**

This study will benefit clinicians who seek to evaluate patients’ levels of exercise adherence and those intending to undertake research, service evaluation, or audit relating to exercise adherence in the musculoskeletal field. The findings will impact upon new research studies which aim to understand the factors that predict adherence with exercise and which test different adherence-enhancing interventions. PROSPERO reference: CRD42013006212

## Background

Musculoskeletal disorders, such as low back pain, shoulder disorders, and osteoarthritis, are common, with estimates suggesting an average prevalence of 38% [1], which increases markedly with age [1] and is likely to continue to rise due to the ageing population and increasingly sedentary lifestyles [2,3]. Musculoskeletal disorders cause more functional limitations than any other group of disorders within the adult population and lead to enormous healthcare expenditure and loss of work [4].

Clinical guidelines advocate the use of exercise programmes for musculoskeletal disorders [5,6]. Exercise can encompass a wide range of interventions such as general (aerobic) exercise, specific body-region exercises for strengthening and flexibility, continuing normal physical activities, and increasing general physical activity levels [7]. Systematic reviews consistently show the beneficial effects of different types of exercise on key clinical outcomes such as pain, physical function, and quality of life [8-10]. Adherence, defined as “the extent to which a person’s behaviour corresponds with agreed recommendations from a healthcare provider”, is considered to be an important pre-requisite for the success of exercise programmes for musculoskeletal disorders [11,12]. Adhering to an exercise programme enhances its effectiveness, and patients who undertake regular physical activity may be less likely to progress to recurrent, persistent, or disabling problems [13,14].

Despite its importance, adherence to clinic-based exercise protocols is often around 50% [15,16] and is usually worse for unsupervised home exercise programmes [17,18]. Non-adherence to exercise recommendations may negatively affect treatment effectiveness, treatment duration, efficiency of use of personnel and equipment, the therapeutic relationship, waiting times, and cost of care [19-21], and may also be responsible for non-significant research outcomes within a clinic-based research context [22]. Although numerous interventions exist that may potentially improve adherence to exercise for musculoskeletal disorders [7,23-25], in order to robustly test the effectiveness of these potential interventions, reliable and reproducible measures of exercise adherence are first required.

Due to the multi-dimensional nature of exercise adherence [26], including completing exercise and physical activity correctly, in different settings and at the agreed ‘dose’, accurate measurement of exercise adherence can be challenging. No gold standard measure of adherence exists [27]. Moreover, the most appropriate measure of adherence for one type of therapeutic exercise (for example specific body-region exercises for strengthening and flexibility) may not be appropriate to measure adherence to other types of therapeutic exercise, such as increasing general physical activity levels. Within clinical practice, more objective measures of exercise adherence, such as a diary, are underutilised [28], and in randomised controlled trials of exercise for musculoskeletal disorders measurement of adherence is either non-existent or limited by use of non-standardised instruments that capture data on only one domain of adherence [7,29].

Although a wide range of performance-based, clinician-reported and patient-reported measures of exercise adherence are available, most lack a clear theoretical underpinning [7,30]. In addition, there is wide variation in the use of these measures within clinical research and routine practice settings, and guidelines or consensus regarding how exercise adherence should be assessed do not exist. Agreement on the most relevant, useful and appropriate approach to assessing exercise adherence is essential if research and clinical audit is to be effective in informing both policy and clinical decision-making [31].

This study will seek to achieve a UK-based consensus on the ‘best’ measures of exercise adherence in terms of quality, acceptability, and usefulness to musculoskeletal research and routine clinical practice.

## Methods/design

There are two key stages to the proposed research. First, a systematic review of the quality and acceptability of measures used to assess exercise adherence; second a consensus meeting of UK stakeholders. The systematic review will be conducted in two phases and reported in accordance with the Preferred Reporting Items for Systematic Reviews and Meta-Analyses (PRISMA) guidelines to ensure robust methodology [32]. Phase one will identify all measures that have been used to assess exercise adherence within a musculoskeletal setting. Phase two will seek to identify published and unpublished evidence of measurement and practical properties of identified measures of exercise adherence. The quality of developmental and evaluative studies will be assessed against the COnsensus-based Standards for the selection of health Measurement Instruments (COSMIN) guidelines [33,34]. A short-list of best quality measures will be produced for consideration during stage two of the study, the consensus meeting, which will inform recommendations on the ‘best’ measures of exercise adherence in terms of quality, acceptability, and usefulness to musculoskeletal research and routine clinical practice.

### Stage 1.1: Systematic review – identification of measures

A search strategy combining title/abstract words and database subject headings relating to exercise adherence and musculoskeletal rehabilitation will be used to locate all measures used to assess exercise adherence in a musculoskeletal context (see Search strategy for phase one of the systematic review), and run in the following databases from their inception: Medline, SPORTDiscus, CINAHL Plus with Fulltext, PsycINFO, AMED, The Cochrane Library (Cochrane Database of Systematic Reviews, Cochrane Cemtral Register of Controlled Clinical Trials, Database of abstracts of reviews of effects, Health Technology Assessment Database, and the NHS economic evaluation database), Embase, and Web of Science. A RefWorks database will be used to manage all references.

#### Search strategy for phase one of the systematic review

The search strategies will use title and abstract words/synonyms and database subject headings (e.g., MeSH) to capture the concept of exercise adherence in the context of musculoskeletal rehabilitation, for adult patients. The strategies will also contain the following exclusions: “cardiac rehabilitation”, “pulmonary rehabilitation”, “neuro* rehabilitation”, “stroke”, “child*”, and “infan*”.

To capture ‘exercise adherence’, title and abstract terms/synonyms and database subject headings for adherence will be combined with those for exercise (listed below) using proximity operators (within 3 words), or AND, as applicable.

Adherence terms: Words in title/abstract: adher*, nonadher*, complian*, noncomplian*, concordan*, cooperat*, co-operat*, uncooperat*, unco-operat*, engag*, disengag*, behaviour#, behavior#. MeSH: “Patient Compliance”.

Exercise terms: Words in title/abstract: activ*, exercis*, physical n3 train*, weight n3 train*, sport#, rehab*, MeSH: “Therapeutic Exercise +”, “Exercise Therapy +”, “Exercise +”, “Physical Activity”, “Motor Activity”, “Exercise Movement Techniques”.

To restrict the results to the context of musculoskeletal rehabilitation, the above searches will be combined (AND) with the following search terms/synonyms and database subject headings:

Musculoskeletal rehabilitation terms: Words in title/abstract: osteopath*, chiropract*, musculoskeletal, msk, physiotherap*, rehabilitat*, osteoarthrit*, spondyl*, osteitis, osteochondritis, arthropathy, bursitis, shoulder impingement, myalgia, lordosis, sacroiliac, sciatica, cervicogenic, dyskinesis, tendinitis, tendinopathy, allodynia, hyperalgesia, subluxation, disc, misalignment, osteopathic lesion, frozen shoulder, degenerative joint disease, muscular n3 pain, back n3 pain, lumbar n3 pain, lumbo* n3 pain, spine n3 pain, spinal n3 pain, neck n3 pain, cervical n3 pain, knee* n3 pain, hips n3 pain, hip n3 pain, shoulder n3 pain, ankle# n3 pain, foot n3 pain, feet n3 pain, elbow# n3 pain, hand# n3 pain, flank pain, buttock pain, joint pain, radicular pain, neuralgia, lumbago, arthralgia, adverse neural tension, muscle tear#, sprain* n5 musc*, strain* n5 musc*. MesH: “Osteopathy”, “Osteopathic Medicine”, “Chiropractic”, “Manipulation, Chiropractic”, “Musculoskeletal Diseases +”, “Sciatica”, “Tendinopathy +”, “Allodynia”, “Hyperalgesia”, “Subluxation”, “Back Pain +”, “Neck Pain”, “Neuralgia +”, “Elbow Pain”, “Arthralgia +”, (“Musculoskeletal System +” AND “Pain +”).

Titles and abstracts of all identified articles will be reviewed for inclusion by two independent reviewers and agreement checked. A third independent reviewer will be used to resolve any differences regarding eligibility. Any primary quantitative study (including clinical trials, observational studies, longitudinal studies, case control studies, and case studies) will be included if they involve: i) adults with musculoskeletal disorders, ii) any therapeutic exercise or physical activity intervention, iii) clearly defined and reproducible measures used to assessed adherence to exercise or activity, including patient-reported or clinician-reported measures or exercise diaries (if converted to an adherence measurement scale), and iv) exercise or activity delivered in any therapeutic setting including inpatient, outpatient, and community settings. Studies will be excluded if they are not written in English, involve i) participants under 18 years of age, ii) participants who have received therapeutic exercise and activity for non-musculoskeletal conditions such as diabetes, asthma, and cancer, or iii) if they include healthy volunteers. Performance measures (i.e., muscle strength and joint range of movement), performance of exercise technique and attendance at sessions are often considered proxy measures of exercise adherence and will therefore be excluded from this review. Following title and abstract screening, full text articles of retained studies will be reviewed for inclusion and adherence measures will be identified. Cleary defined and reproducible measures of exercise adherence used within the musculoskeletal field will then be located and collated.

### Stage 1.2: Systematic review – identification of development and/or evaluative papers

Articles reporting the measurement and/or practical properties of identified measures used to assess exercise adherence will be sought by performing further specific searches in the databases identified above for each identified measure. A highly sensitive search filter developed by Terwee et al. [35] for finding studies on measurement properties of measurement instruments will be applied for larger result sets. Authors identified as the first contact for the development of a specific measure will be contacted to locate additional published or unpublished studies that may provide additional evidence of measurement and/or practical properties for the specific measure. Published articles will be included if they provide evidence of development and/or evaluations for clearly defined and reproducible measures used to assess exercise adherence, and published in the English language. We will seek evidence from both within, and outside of the musculoskeletal setting. All titles and abstracts, and where applicable full text articles, will be assessed for inclusion by two independent reviewers and a third reviewer will resolve any disagreements. Reference lists of included articles will be reviewed for additional published articles.

#### Data extraction and appraisal

A data extraction form informed by earlier reviews [36] and the requirements of the COSMIN checklist [33,34] will be used to ensure that data necessary to support an evaluation of both study and measure quality is extracted.

Data extraction will capture study-specific information (including population, intervention, and setting) and measurement tool-specific information. Extraction for measurement properties will seek evidence of reliability (internal consistency, test-retest, intra- or inter-tester, measurement error); validity (content, construct including internal (within scale) analyses and analyses against external criteria, convergent/divergent, and known group differences); evidence of explicit hypothesis testing will be detailed; evidence of the conceptual underpinning and the aspects of exercise adherence which the measure purports to assess will be sought; responsiveness (criterion-based or construct-based assessment); interpretation (minimal important difference); and precision (data quality, end effects). Extraction for evidence of practical properties will include acceptability (relevance and respondent burden) and feasibility. The extent of patient involvement in measurement development and/or application will also be sought.

In accordance with the COSMIN checklist, each measurement property reported by the study will be rated on a 4-point scale (i.e., excellent, good, fair, poor) [33,34]. Study methodological quality will be evaluated per measurement property and determined by the lowest checklist rating [33,34]. Two reviewers will independently undertake data extraction and apply the checklist to each included study. Consensus will be sought through discussion; any disagreements will be resolved using a third reviewer.

#### Data synthesis

As reported in other reviews [37,38], all data will be qualitatively synthesised to determine the overall quality and acceptability of each reviewed measure. The synthesis will take the following factors into account: i) study methodological quality (COSMIN scores); ii) the number of studies reporting specific evidence per measure; iii) the results for each measurement/practical property per measure; and iv) consistency between studies [38]. The data synthesis score will have two elements. First, the overall quality of a measurement property will be reported as: adequate (+), not adequate (−), conflicting (+/−), or unclear (?). Second, levels of evidence for the overall quality of each measurement property will be further defined to indicate ‘strong’, ‘moderate’, ‘limited’, ‘conflicting’, or ‘unknown’ evidence as detailed by Elbers et al. [38]. The synthesis will produce a shortlist of ‘best’ quality measures that will be further considered in stage two.

### Stage 2: Consensus meeting

The final stage of the project will be a one-day ‘expert’ consensus meeting with 15 participants representing key stakeholders, including lay representatives (n = 6), clinicians who use therapeutic exercise (n = 3), expert researchers in the field of adherence or exercise therapy (n = 3), and service managers (n = 3). We will reach a consensus on the ‘best’ measures of exercise adherence in terms of quality, relevance, acceptability, feasibility, and appropriateness for musculoskeletal healthcare in both clinical research and routine practice settings. The strengths and limitations of reviewed measures, and a future research agenda will be produced.

A structured group decision-making approach, or Nominal Group Technique [39-41], will be used to work towards consensus on two main questions:

1. What should be measured when assessing exercise adherence?

2. How should exercise adherence be measured and how ‘useful’ are the shortlisted ‘best’ measures of exercise adherence with respect to:

•Relevance (to the aspects of exercise adherence viewed as most important);

•Acceptability (to patients who are required to adhere to exercise regimes);

•Appropriateness (to the musculoskeletal population)

•Feasibility (for use in clinical research and/or routine practice settings)?

Relevant research will be summarised and sent to participants in advance of the meeting. This will include i) a synthesis of the systematic review and copies of the ‘shortlisted’ measures (how is adherence currently measured); and ii) a list of aspects, or domains, of exercise adherence currently assessed in published research (what aspects of adherence are currently measured).

Participants will also receive a questionnaire with two key sections on what and how to measure exercise adherence. Participants will be asked to rate the relative importance of each aspect, or domain of exercise adherence identified in the systematic review. Importance ratings will be made on a 9-point GRADE scale (1 to 3 = not important; 4 to 6 = important; 7 to 9 = critical) [42,43]. Importance will be defined as ‘how important is it that this aspect, or domain, of adherence is included in the assessment of exercise adherence?’ Participants will also be asked to consider the relevance and feasibility of each measure for research or clinical musculoskeletal settings. Measures will be rated on separate 9-point GRADE scales (1 to 3 = not relevant/not feasible; 4 to 6 = relevant/feasible; 7 to 9 = most relevant/highly feasible). Finally, participants will be asked to rate the suitability of each measure for the assessment of exercise adherence in i) research (yes/no) or ii) routine practice (yes/no) musculoskeletal settings.

The questionnaire content will be piloted with representative stakeholders (n = 3; to include a patient, physiotherapist, methodologist/researcher). Participants will be asked to return all completed questionnaires in advance of the meeting to allow results to be collated. Where appropriate, participants will be encouraged to provide additional comments or contributions to the candidate lists of domains and outcome measures for further discussion at the meeting.

The consensus meeting will be structured into three discrete sections [44-46]. First the evidence synthesis will be re-presented [43], and group results from postal completion of the nominal group questionnaire shared with the group. Individual participants will also receive their own individual scores, supporting comparison with the wider group. Next, semi-structured group discussions will be facilitated and participants will again be invited to address the two core questions stated above. Finally, a plenary session will be convened and the results from the group sessions fed back to all participants. There will be an anonymised voting process, during which we will converge on a common view of what aspects of exercise adherence should be assessed (1. What to measure?), and make recommendations for the most relevant and appropriate method of assessment (2. How to measure?). During the final vote participants will be invited to vote as to whether each domain (yes/no) and each outcome measure (yes/no) should be included in the assessment of exercise adherence. The meeting will seek to make clear recommendations for simple, relevant, and appropriate assessment of exercise adherence both within research and clinical practice settings within the musculoskeletal field.

## Discussion

Adherence to therapeutic exercise is a pre-requisite for successful rehabilitation, yet its accurate measurement is challenging [11]. In clinical practice, more objective measures of exercise adherence are underutilised [28]. In clinical trials of exercise for musculoskeletal pain, adherence is not always measured, and when it is, the measures used are often not validated or standardised, making it difficult to compare the effectiveness of different interventions and impossible to pool data in the form of a meta-analyses [7,29]. This review will identify, summarise, and critically evaluate available measures of exercise adherence and develop recommendations about the most promising measures which are relevant for musculoskeletal research and clinical practice. This will benefit clinicians evaluating patients’ levels of exercise adherence and those intending to undertake research, service evaluation, or audit relating to exercise adherence in the musculoskeletal field. The findings will impact upon new research studies which aim to understand the factors that predict adherence with exercise, which test different adherence-enhancing interventions.

## Abbreviations

AMED: Allied and complementary medicine; CINAHL: Cumulative index to nursing and allied health literature; COSMIN: COnsensus-based Standards for the selection of health Measurement Instruments; GRADE: Grading of recommendations assessment development and evaluation; PRISMA: Preferred reporting items for systematic reviews and meta-analyses; UK: United Kingdom.

## Competing interests

The authors declare that they have no competing interests.

## Authors’ contributions

All authors have made substantial contributions to the conception and design of the study and participated in writing the grant application. MH drafted the manuscript. SM, KH, TP, and MG edited and finalised the manuscript. All authors read and approved the final manuscript.
